# Dahuang—Taoren, a botanical drug combination, ameliorates adenomyosis *via* inhibiting Rho GTPases

**DOI:** 10.3389/fphar.2023.1089004

**Published:** 2023-03-06

**Authors:** Ya Lei, Xianyun Fu, Minmin Chen, Yongli Yi, Ping Mao, Li Peng, Zhao Qu

**Affiliations:** ^1^ The First College of Clinical Medical Science, China Three Gorges University, Yichang Central People’s Hospital, Yichang, China; ^2^ Third-Grade Pharmacological Laboratory on Chinese Medicine Approved by State Administration of Traditional Chinese Medicine, Medical College, China Three Gorges University, Yichang, China

**Keywords:** adenomyosis, dahuang -taoren combination (DT), RhoA, Cdc42, ROCK1, Rac1

## Abstract

**Introduction:** Dahuang-Taoren (DT) is a classic combination of botanical drugs applied to treat pain-related diseases in ancient China. Today, DT is frequently applied for dysmenorrhea of adenomyosis (AM) in the clinic. Growing evidence indicates Rho GTPases may play an essential role in AM progression. However, the potential mechanism of DT on Rho GTPases in AM remains unclear.

**Methods:** The expressions of Rho GTPases in the patients with AM were evaluated. Further, pituitary transplantation-induced AM mice and the primary AM endometrial stromal cells (AMESCs) were subjected to DT intervention.

**Results:** The results revealed that the expressions of Rho GTPases were significantly upregulated in both AM patients and AM mice. The DT could reduce pathological infiltration, relieve hyperalgesia, and alleviate cytoskeleton remodeling in AM mice. Besides, the migration and invasion of AMESCs were markedly inhibited after exposure to DT.

**Discussion:** These effects may be linked to the decreased Rho GTPases expression. The results may offer a novel explanation of DT against AM.

## 1 Introduction

Adenomyosis (AM) has emerged as one of the major concerns threatening to the physical and mental health of reproductive-age women (Alcalde et al., 2021). Refractory dysmenorrhea and chronic pelvic pain in AM represent a challenge for medical support. The development of more efficacious, non-hormonal therapeutics for AM is still an unmet medical need begging to be fulfilled (Nirgianakis et al., 2021; Fan et al., 2022).

AM is the presence of endometrial tissues with tumor-like features in uterine smooth muscle layer locations. Rho GTPases, a kind of regulator for intracellular actin dynamics, mainly consisting of RhoA, Rock1/2, Cdc42, and Rac1, can dramatically interfere with the motility of tumor cells (Wen et al., 2017). Growing evidence indicates that endometriotic lesions have malignant invasion ability, and, as such, Rho GTPases may play an essential role in AM progression. The increased expressions of RhoA and Rock1/2 have been found in AM, which may be responsible for the enhanced invasion of the endometrium into the myometrium (Zhai et al., 2020). Other researchers indicated an abnormal expression of Cdc42 in the eutopic endometrium of AM (Goteri et al., 2006). Further studies verified that the expressions of Rho GTPases were positively associated with the severity of menorrhagia and dysmenorrhea in AM (Jiang et al., 2018) (Wang et al., 2016). Thus, inhibiting Rho GTPases may be a promising treatment option for AM.

In China, traditional Chinese medicine (TCM) is widely used to treat AM. Blood stasis syndrome is considered the core pathological process of pain in TCM, thus, promoting blood circulation and removing stasis is an essential treatment for pain-related disorders, including AM. The data mining of TCM showed that promoting blood circulation and removing blood stasis exhibited therapeutic effects on AM, significantly relieving symptoms of dysmenorrhea (Bi, 2020). Dahuang-Taoren (DT), a classic combination of botanical drugs, originating from Jin-Gui-Yao-Lue (Synopsis of Prescriptions of the Golden Chamber) in the Han dynasty (Xu et al., 2022), contains two herbs: Rhubarb (the dried rhizomes of *Rheum palmatum* L.) and Peach kernel (the dried mature seed of *Prunus persica* L.) Batsch). The main function of Rhubarb (Dahaung) and Peach kernel (Taoren) in TCM theory is as a potent purgative to free the bowels, remove excess heat, and promote blood flow to resolve stasis. Several studies on AM have demonstrated that dysmenorrhea was effectively attenuated by TCM recipes containing DT (Guan et al., 2014) (Ye et al., 2020). However, the therapeutic mechanisms of DT on Rho GTPases remain poorly understood.

In China, TCM doctors prescribe DT in the form of extract granules instead of herbal decoctions because the extract granules produced by good manufacturing practice manufacturers are quality assurance and easy to take. In this study, the potential role of Rho GTPases was revealed through *in vitro* and *in vivo* experiments, and the underlying regulation of DT extract granules on AM was also investigated.

## 2 Materials and methods

### 2.1 DT extract granules

The DT granules were the extract of dried rhizomes of *R. palmatum* L. [Polygonaceae] (Dahuang, Rhubarb) and the dried mature seed of *P. persica* L.) Batsch [Polygonaceae] (Taoren, Peach kernel), which were manufactured by Beijing Kangrentang Pharmaceutical Co., Ltd. (Beijing, China) with standardized quality control. The specific preparation method of DT is described in the Supplementary Material, and the batch numbers of DT extract granules during the study period were also recorded (Supplementary Material S1). The quality control of the DT extract granules was confirmed by high-performance liquid chromatography (HPLC) (Supplementary Material S1).

### 2.2 Reagents and antibodies

FITC-conjugated donkey anti-rabbit IgG antibody (cat. No. 02–18-06) was obtained from SeraCare Life Sciences. Inc. (Massachusetts, United States). RhoA (cat. No. 10749-1-AP), Cdc42 (cat. No. 10155-1-AP), Rac1 (cat. No. 24072-1-AP), and Rock1 (cat. No. 21850-1-AP) antibodies were supplied by Proteintech Group, Inc. (Wuhan, China). GAPDH (cat. No. GB11001) and vimentin (cat. No. GB111308), CK18 (cat. No. GB11232), and HRP labeled goat anti-rabbit Ig G antibody (cat. No. GB23303) were provided by Service Technology Co., Ltd. (Wuhan, China). Fetal bovine serum (FBS) (Lot no. 13011–8611) was offered by Tianhang Biotechnology Co., Ltd. (Zhejiang, China). Dulbecco’s Modified Eagle Medium/Nutrient Mixture F-12 medium (cat. No. PYG0074) was purchased from Boster Biological Technology Co., Ltd. (Wuhan, China).

### 2.3 Clinical samples

Twelve patients undergoing the hysterectomy were recruited from Affiliated First People’s Hospital of China Three Gorges University from September 2021 to July 2022. Among them, six patients with AM confirmed by pathological biopsy were recruited as the model group, while another six patients with uterine fibroids were enrolled as the control group. The eutopic endometrial tissues were collected from the patients during the hysterectomy. This study was approved by the Institutional Review Board of China Three Gorges University (Approval NO. 2022CA002).

### 2.4 Isolation and culture of primary cell

The eutopic endometrial tissue fragments were digested with 0.2% type Ⅳ collagenase in an electric-heated thermostatic water bath. The debris and epithelial cells were removed by 100 μm and 40 μm cell strainer to obtain AM endometrial stromal cells (AMESCs). AMESCs collected by centrifuging at 1,000 rpm for 5 min were maintained in DMEM/F12 medium with 15% FBS in humidified 5% CO_2_ at 37 °C. 3 or 4 passages of cells passaged at a ratio of 1:2 were selected for the following experiments.

### 2.5 Cell identification assay

Immunofluorescent staining was used to identify the AMESCs. Firstly, AMESCs were seeded onto the coverslips and fixed with 4% paraformaldehyde. Further, cells were permeated with 0.1% Triton-X--100, blocked with 3% bovine serum albumin and incubated overnight at 4 °C with primary antibodies (vimentin (1:200), ck18 (1:3000)). FITC-conjugated donkey anti-rabbit IgG antibody was added for 10 min incubation at room temperature. In the end, the nuclei were counterstained with DAPI solution and observed by a fluorescence microscope.

### 2.6 Cell viability assay

MTT assay was performed to evaluate the cell viability after DT treatment. Specifically, AMESCs were seeded at 5 × 10^4^cells/well in 96-well plates within DMEM/F12 medium with 15% FBS for 48 h and then treated with DT at different concentrations (0, 0.125, 0.25, 0.5, 0.75, 1, and 1.25 mg/mL) for 48 h, followed the addition of 10 μL of 5 mg/mL MTT for 4 h at 37°C. Subsequently, the supernatant was aspirated and discarded, and 150 µL of DMSO was added to each well for 15 min. The optical density of each well was determined at a wavelength of 490 nm under a microplate analyzer to assess the remaining number of survival cells.

### 2.7 Wound healing assay

The AMESCs were inoculated into 6-well plates in a medium containing 15% FBS in a constant temperature incubator at 37°C and 5% CO_2_. After cell covering the bottom of the wells, culture plates were performed to scratch with a sterile 10 µL pipette tip to create a homogenous wound. Then the medium was replaced according to the groups: control group, 0.125 mg/mL DT, 0.25 mg/mL DT, and 0.5 mg/mL DT. The exact position wound images were captured at 0, 12, and 24 h. After that, ImageJ 1.8.0 software was used to measure the wound healing areas at each time point, and the ability of AMESCs migration was evaluated by the percentage of wound healing areas to initial area at 12 and 24 h.

### 2.8 Animals study

Female ICR mice weighing 18–22 g were purchased from the Beijing Weitong Lihua Laboratory Animal Technology Co., Ltd. (Beijing, China) and maintained under specific pathogen-free (SPF) conditions. All animals received human care, and experiment procedures conformed to the guidelines approved according to the Animal Experiment Ethics Committee of the Medical College of China Three Gorges University (Approval NO. 20220101).

Random allocation was performed to divide thirty mice into three groups: control (n = 10), model (n = 10), and DT (n = 10). The mice of the model and DT group were anesthetized with disoprofol (Xian Nippon Pharmaceutical Co. Ltd. Xian, China, No. H19990282, 100 mg/kg) and then received allograft pituitary transplantation. In short, pituitary suspensions obtained from age-matched male mice were injected into the right uterus of female mice. After intraperitoneal infusion of gentamycin solution (0.25 mL, 20,000 units/20 g), the abdomen was closed by the conventional method. Subsequently, the optimal dosage and ratio of TCM pairs were definitized according to ancient documents mining and early animal experiments (Wang L D et al., 2018). Among these, the mice of DT groups were fed a DT-dissolved solution (0.3125 g/kg) and repeatedly administered every 24 h for 8 weeks, while other groups received the same amount of normal saline. The determination of drug dosage is of therapeutic relevance and has been demonstrated by our preliminary research (Fu et al., 2022; Yi et al., 2022). After the end of the gavage period, all mice were humanely killed by decapitation, and mice uterus were obtained.

### 2.9 Behavior test

Behavioral tests were conducted to detect the effect of DT intervention. The pain behavior of each group was measured before and after modeling.

Determination of heat hyperalgesia: After heat stimulation of the hind plantar of mice with a metal plate with a surface capable of being heated to a constant temperature of 50.0 (±0.1)°C, the reaction time of the hind claw retraction was recorded to evaluate paw sensitivity to pain.

Determination of mechanical hyperalgesia: The hind plantar of mice was stimulated with the mechanical ciliary filament of the von Frey apparatus. At the same time, the reaction time of hind claw retraction was recorded to evaluate paw sensitivity to pain.

### 2.10 Hematoxylin and eosin (HE) staining

Mice uterine tissues were fixed in 4% paraformaldehyde, extracted in paraffin-embedded with xylene, and cut into 5 µm-thick sections. The sections were dewaxed by xylene and hydrated in a series of ethanol concentrations, followed by reacting with hematoxylin and eosin to determine pathological changes. Images were observed and photographed under an optical microscope, and the distribution of ectopic endometrial cells in the uterus was employed to describe the degree of histopathological injury.

### 2.11 Transmission electron microscopy

The isolated uterine tissues, 1 mm^3^ in size, were fixed with 2.5% glutaraldehyde and 1% osmic acid, washed twice with PBS, and dehydrated in a series of ethanol concentrations at room temperature. After embedding and solidifying, these samples were sectioned into 70 nm-thick slices using an ultramicrotome and electron stained by lead citrate. The ultrastructural morphology of the cytoskeleton was visualized and photographed under the transmission electron microscope.

### 2.12 Immunofluorescence staining

Frozen uterus tissues of each group were cut into 5-µm slides. Furthermore, the sections were dewaxed, rehydrated, antigen-repaired, and sealed in the 5% bovine serum albumin. The Sections were specimens incubated with the corresponding primary antibody at 4 C overnight and followed by incubation with goat antirabbit FITC-linked antibody at 37°C for 30 min. DAPI was added to counterstain the nuclei, followed by sealing with an anti-fluorescence quenching agent. An inverted fluorescence microscope examined the staining results, and the software of image-Pro Plus counted the average fluorescence intensity. Red and Greens staining was considered positive.

### 2.13 Western blotting analysis

Total proteins were harvested using lysis buffer from clinical eutopic endometrial tissues and AMESCs. BCA protein assay was allowed to detect the protein concentration following the manufacturer’s instructions. The proteins were subjected to 10% sodium dodecyl sulfate-polyacrylamide gel electrophoresis, and the voltage of concentrate glue and separation glue was set at 80 V and 110 V, respectively. PVDF membranes were subsequently coated with total proteins and incubated overnight at 4°C with the corresponding primary antibody (GAPDH (1:2000), RhoA (1:2000), Rock1 (1:3000), Cdc42(1:2000), Rac1 (1:1,000)) at 4°C overnight, respectively. And then, incubation of HRP-linked secondary antibody was carried out for 1 hour. Proteins bands were visualized using an ECL substrate, images were scanned with a chemiluminescence imaging system, and semi-quantitatively analyzed using ImageJ 1.8.0.

### 2.14 Statistical analysis

All fluorescence images were analyzed by image-Pro Plus software, and all statistical analyses were performed using the SPSS 23.0 statistical software (IBM Corp, Armonk, NY, United States). Measurement data results were expressed as means ± SD. One-way ANOVA was used to compare multiple groups, and an independent sample *t*-test was used to analyze the differences between the two groups. A value of *p < 0.05, **p < 0.01, ***p < 0.001 were considered to be statistically significant.

## 3 Results

### 3.1 Expression of Rho GTPases was upregulated in eutopic endometrial tissues of AM patients

**FIGURE 1:**
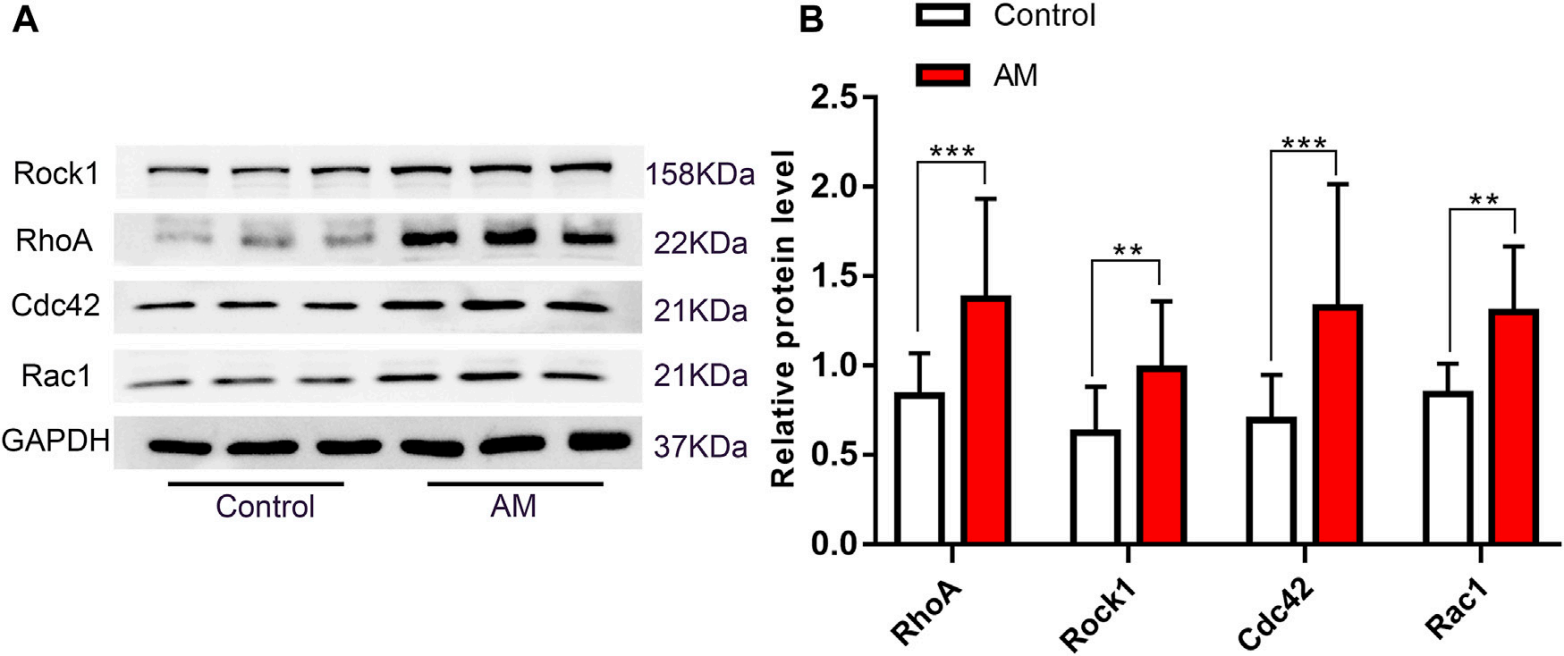
Expression of Rho GTPases in uterine tissues. **(A)** Western blotting of RhoA, Rock1, Cdc42, Rac1. **(B)** Quantitative analysis of RhoA, Rock1, Cdc42, and Rac1. Data were expressed as means ± SD, **p < 0.01, ***p < 0.001 versus the Control group.

As shown in Figures 1A, B, all the protein expressions of Rho GTPases were abnormally changed. The protein levels of RhoA, Rock1, Cdc42, and Rac1 were significantly elevated compared with the control group in eutopic endometria.

**FIGURE 2:**
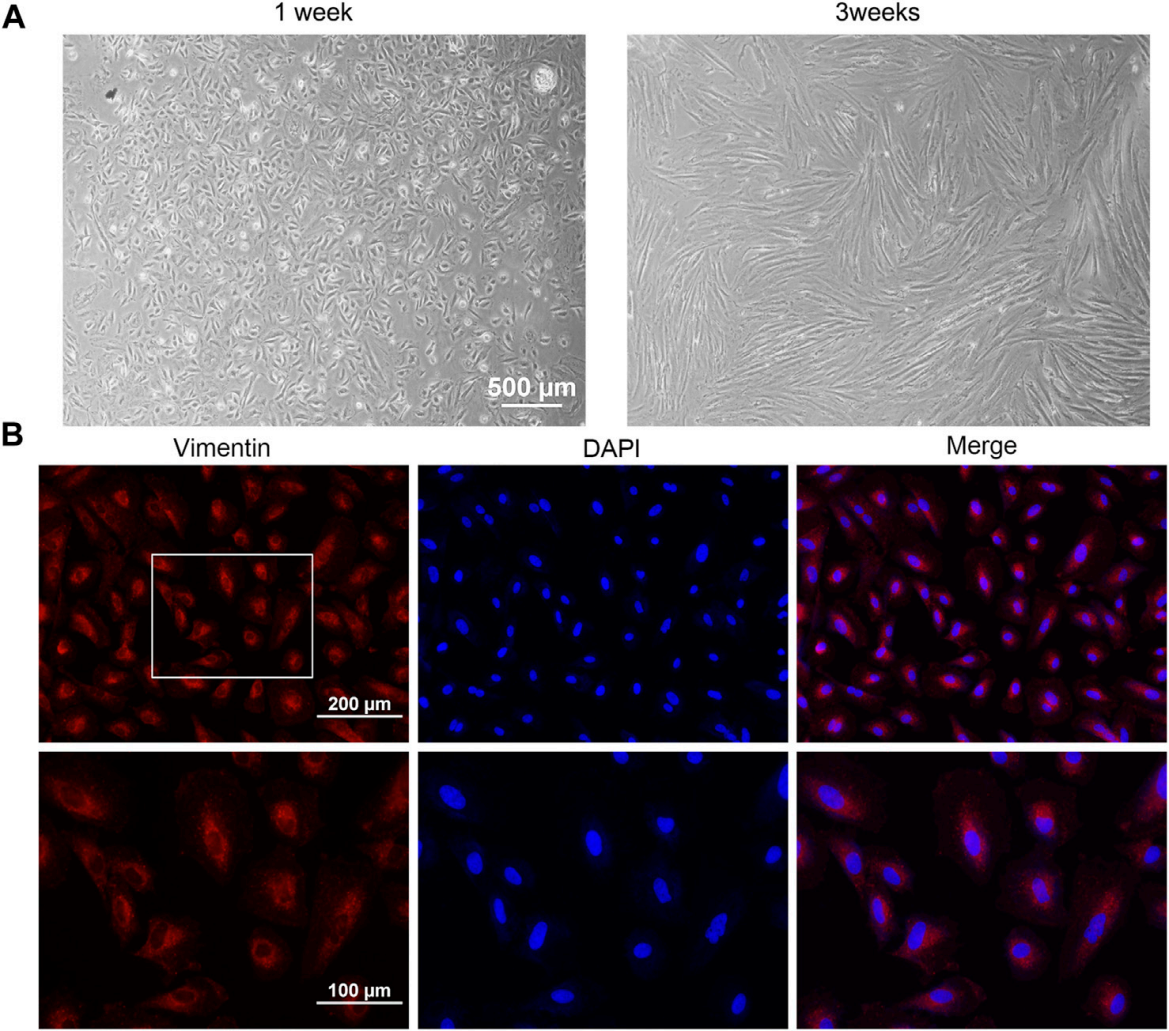
Cell culture and identification. **(A)** Cell morphology of AMESCs. **(B)** Cell identification assay of Vimentin (Red) and DAPI (blue).

To further assess the effect of Rho GTPase, primary AM endometrial stromal cells were successfully isolated and cultured *in vitro*. The AMESCs were presented as large, spindle, or star-shaped, with large oval nuclei observed under bright field micros (Figure 2A). Immunofluorescent staining results showed that the expression of vimentin staining was positive (red) and CK18 staining was negative (green), and the cell purification rate was more than 90% (Figure 2B).

### 3.2 DT inhibited AMESCs viability and migration

**FIGURE 3:**
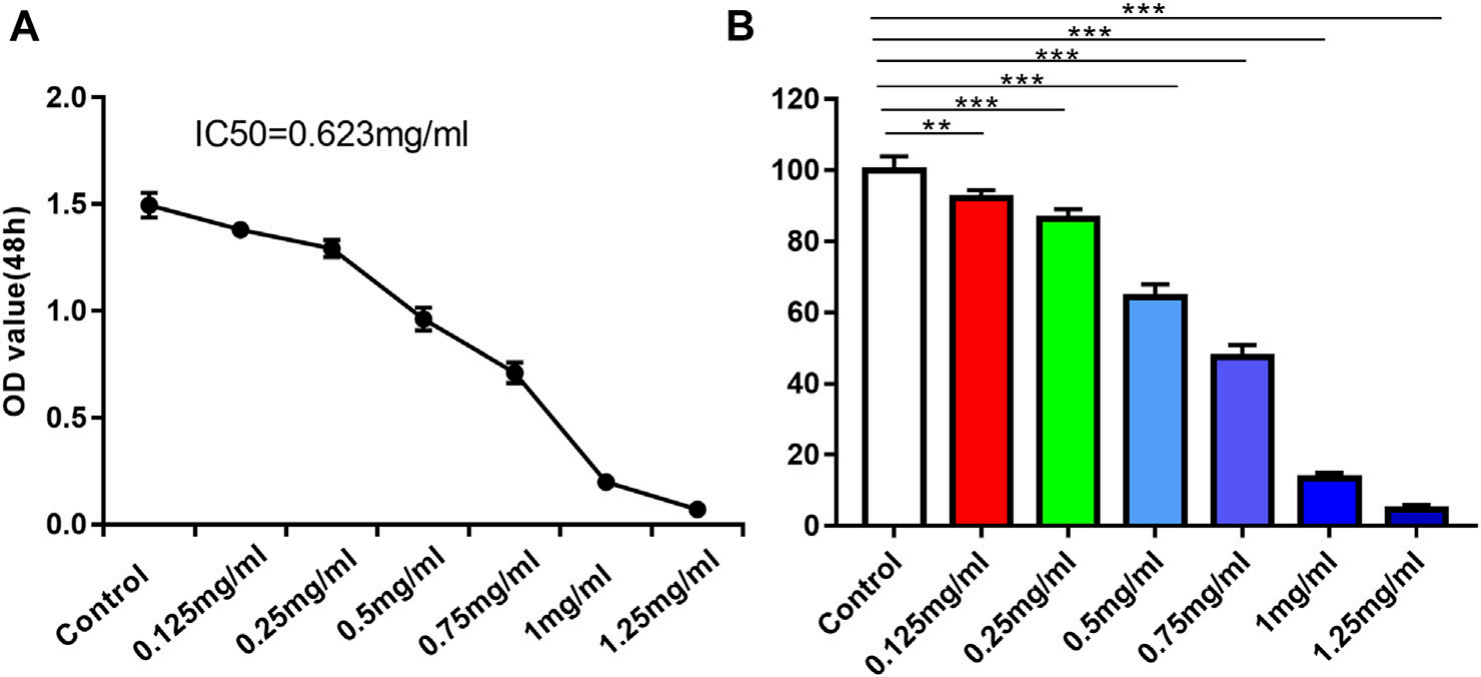
**(A)** IC50 line diagram of DT inhibition of AMSECs cells. **(B)** DT significantly suppressed AMESCs proliferation. Data were expressed as means ± SD, **p < 0.01, ***p < 0.001 versus the Control group.

To evaluate the effect of DT on the viability of AMESCs, we tested the cell viability by MTT assay. AMESCs were treated with a series of concentrations of DT diluent (0, 0.125, 0.25, 0.5, 0.75, 1 and 1.25 mg/mL) for 48 h. At concentrations greater than or equal to 0.125 mg/mL, a noticeable difference was found between the DT treatment group and the control group (Figure 3B). After extending the observation time, a dose-dependent relationship was observed between cell viability and the concentration of DT. As shown in Figure 3A, the survival rate of cells was approximately 50% when they were cultured with the 0.623 mg/mL DT at 48 h.

**FIGURE 4:**
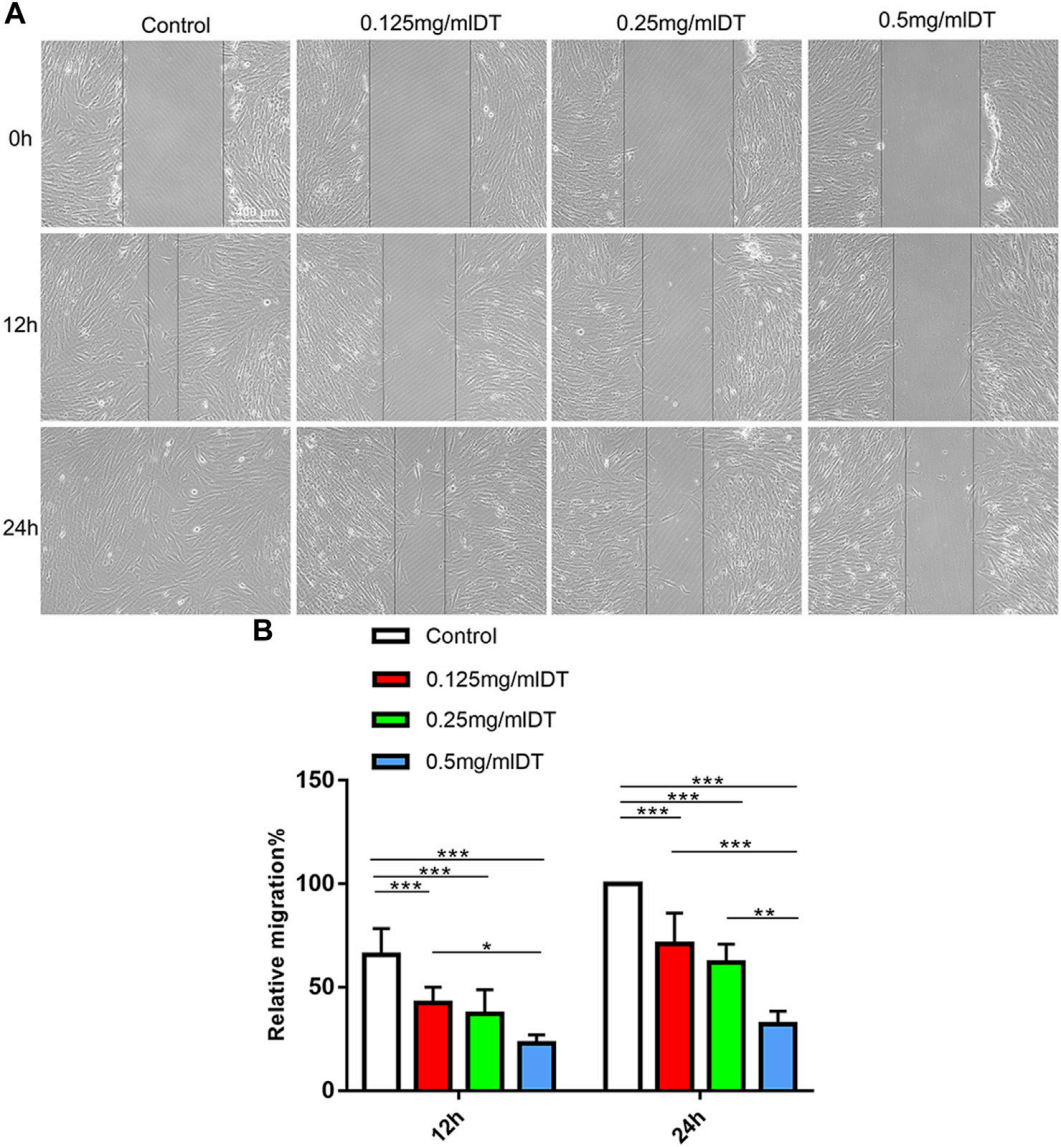
Cell migration. **(A)** Wound Healing Assay was used to analyze the ability of cell migration. **(B)** Quantitative analysis of relative migration. Data were expressed as means ± SD, *p < 0.05, **p < 0.01, ***p < 0.001.

Wound closure assays further evaluated the impact of DT on the migration capacities of AMESCs. AMESCs were treated with control, 0.125 mg/mL, 0.25 mg/mL, and 0.5 mg/mL of DT 12–24 h (Figure 4A). The scratched areas of control were declined compared with DT treatment (Figure 4B). And after prolonging the diving time, the movement velocity of 0.25 mg/mL DT and 0.5 mg/mL were significantly restrained.

### 3.3 DT modulated the expression of RhoA, Rock1, Cdc42 and Rac1

**FIGURE 5:**
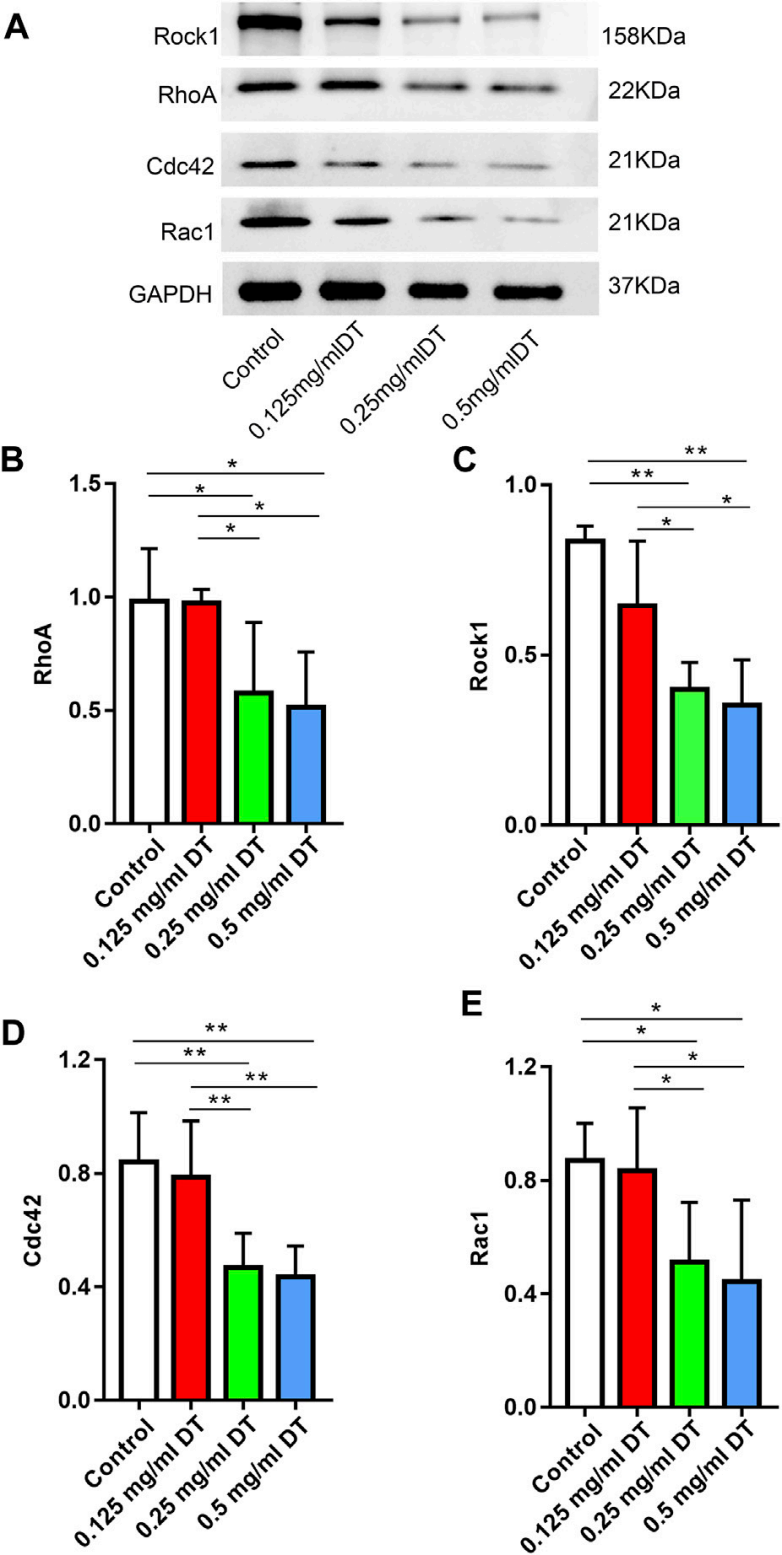
**(A)** Western blotting assay analyzed the levels of RhoA, Rock1, Cdc42, and Rac1 in AMESCs incubated with control, 0.125 mg/mL DT, 0.25 mg/mL DT, and 0.5 mg/mL DT. **(B–E)** Quantitative analysis of relative protein levels. Data were expressed as means ± SD, *p < 0.05, **p < 0.01.

According to the western blotting test, the expressions of RhoA, Rock1, Cdc42 and Rac1 were markedly downregulated in 0.25 mg/mL and 0.5 mg/mL groups compared with control and 0.125 mg/mL DT in a dose-dependent manner, which indicated that DT might affect the process of AMESCs proliferation and migration by Rho GTPases (Figure 5).

### 3.4 DT enhanced sensitivity to pain in mice with AM

**FIGURE 6:**
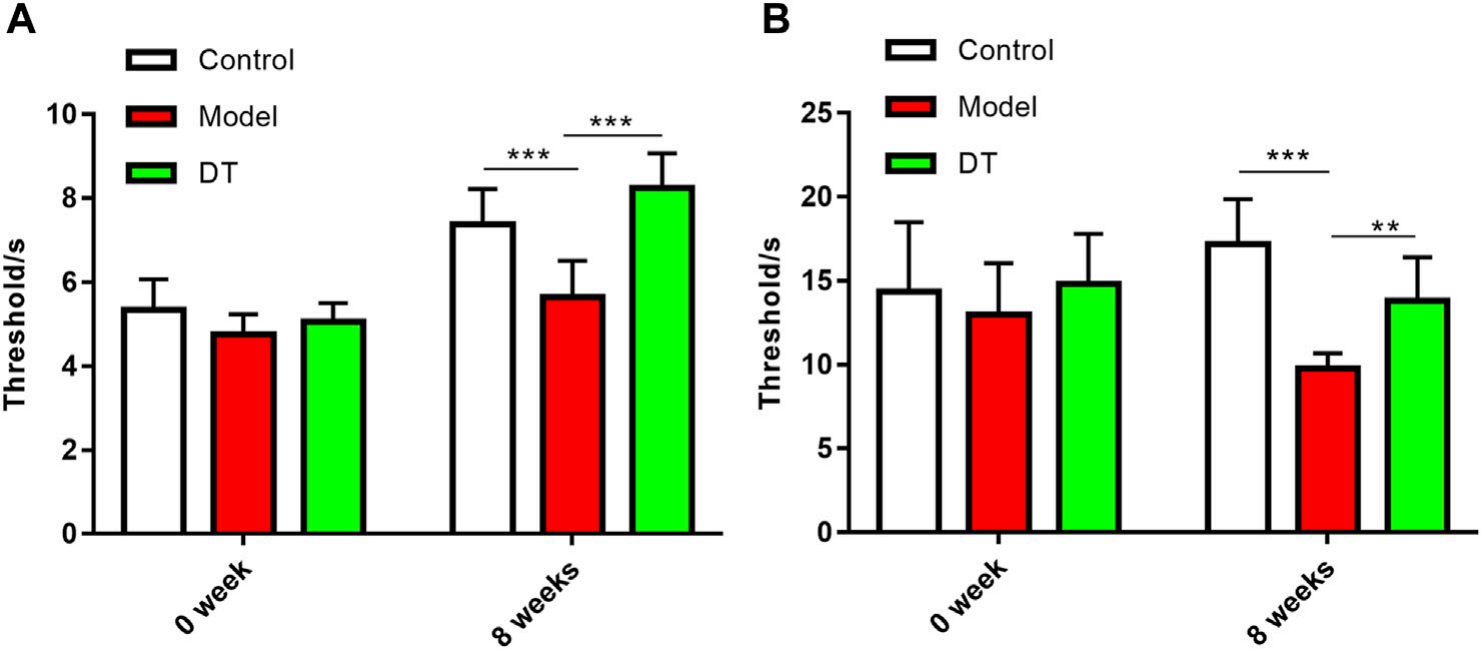
DT improved the sensitivity to pain in AM mice. **(A)** Mechanical hyperalgesia. **(B)** Thermal hyperalgesia. Data were expressed as means ± SD, **p < 0.01, ***p < 0.001.

The sensitivity to pain in mice with AM was measured in two different means, presented as claw retraction time. A lower pain threshold indicates that the mice are more sensitive to pain. As shown in Figure 6, 8 weeks after the intervention of heat and mechanical hyperalgesia, the pain threshold of the mice in the model group was significantly lower than other two groups (*p* < 0.05), demonstrating that the mice in the model group were more sensitive to pain and the symptom was alleviated by DT.

### 3.5 Improvement of DT on pathological infiltration

**FIGURE 7:**
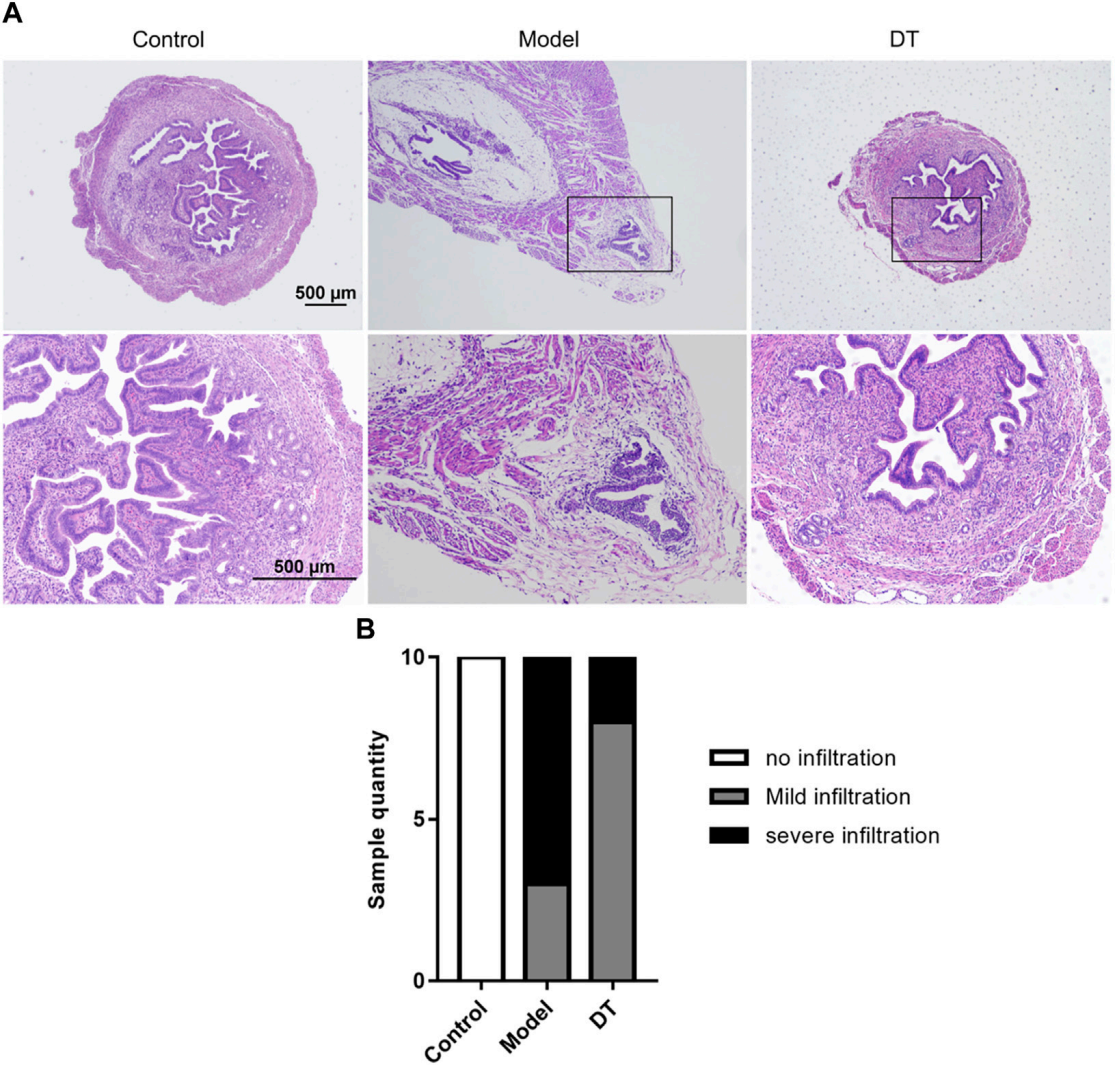
**(A)** Representative microscopic images of the uterus of normal mice (Control) and AM mice treated (DT) and untreated with DT (Model). **(B)** The number of mice quantified the representative relative degree of infiltration.

HE staining showed that the AM mouse model was successfully established (Figure 7A). The infiltration degree of the endometrial invasion was reduced after DT treatment (Figure 7B).

### 3.6 DT alleviated the cytoskeleton remodeling of the endometrium muscular junction (EMI)

**FIGURE 8:**
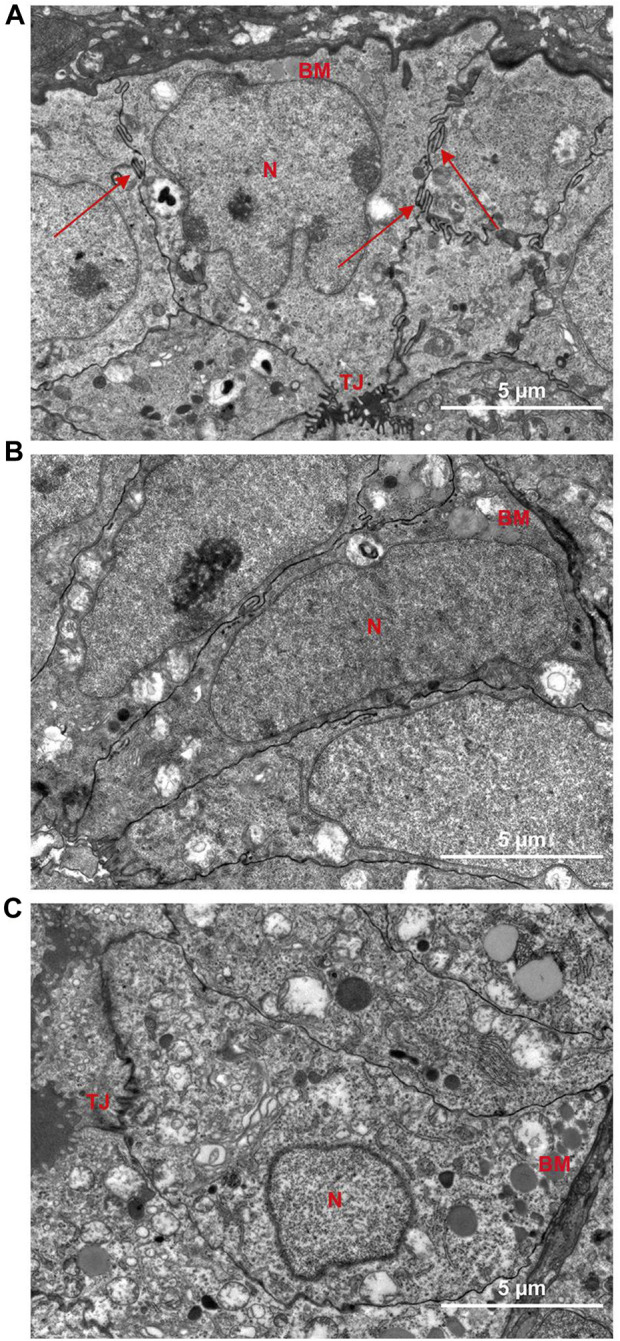
Representative electron microscopy images of the uterus of Control group **(A)** and Model group **(B)** and DT treatment group **(C)**. N, nuclei; BM, basement membrane; TJ, tight junction; Red arrows, finger-like protrusion.

Transmission electron microscopy results demonstrated that the glandular epithelial cells in the control group were columnar, and the surface of the cell membrane represented finger-like structures (Red arrows) (Figure 8A). Multiple tight junctions (TJ) were observed and established an interwoven network to strengthen the toughness and mechanical support between their cells. The glandular epithelial cells from the model group were elongated to fusiform, followed by the decrease of TJ and the degradation of finger-like structures (Figure 8B). After DT treatment (Figure 8C), the cells were restored to a columnar arrangement, and part of TJs could be identified.

### 3.7 DT reduced expression of RhoA, Rock1, Cdc42, and Rac1 in mice eutopic endometrial tissues

**FIGURE 9:**
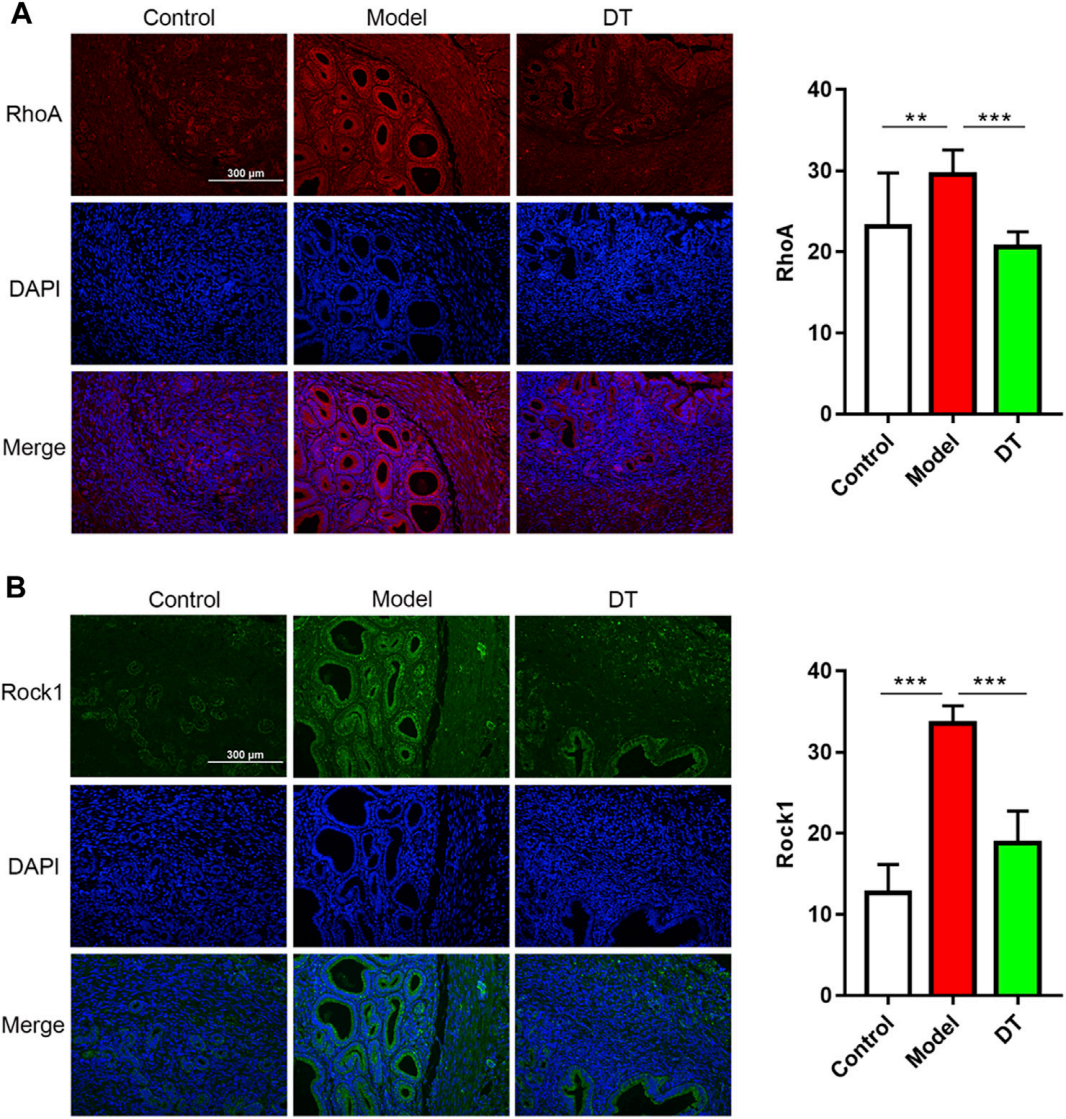
Expression of Rho GTPases in mouse uterine tissues. Immunofluorescence assay of RhoA **(A)**, red and Rock1 **(B)**, green and quantitative analysis of RhoA and Rock1. Data were expressed as means ± SD, **p < 0.01, ***p < 0.001 versus the model group.

**FIGURE 10:**
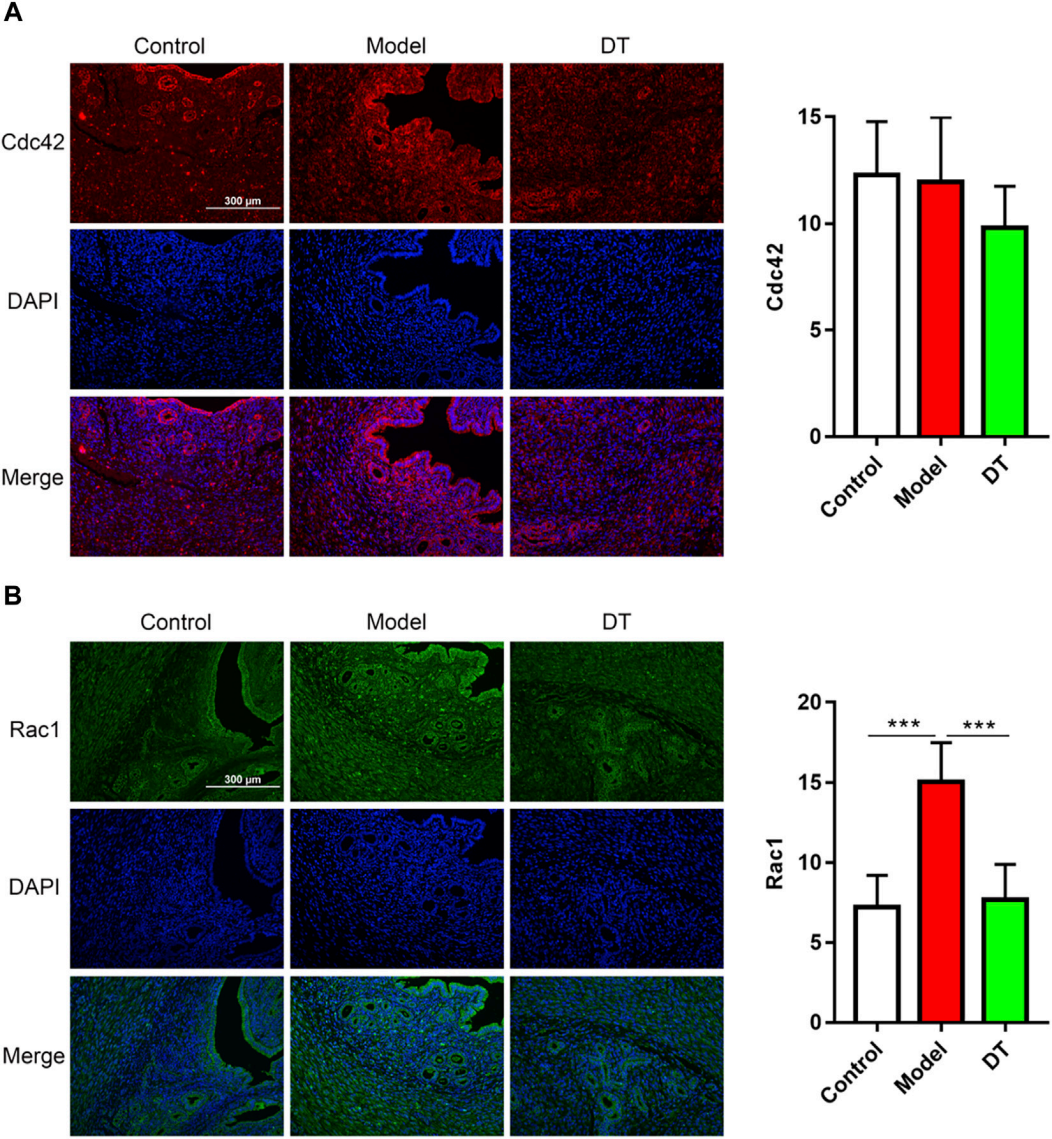
Immunofluorescence assay of Cdc42 **(A)**, red and Rac1 **(B)**, green and quantitative analysis of Cdc42 and Rac1. Data were expressed as means ± SD, ***p < 0.001 versus the model group.

The immunofluorescence assay results demonstrated that there were spots of the RhoA, Rock1, Cdc42, and Rac1 expressions in endometrial and muscular (Figures 9, 10). Compared with the control group, there were more RhoA, Rock1, and Rac1 expressions in mice with AM from the model group, and a noticeable difference was observed. Furthermore, RhoA, Rock1, and Rac1 expression levels were considerably reduced following DT treatment, indicating that DT can hinder cytoskeleton reconstruction. DT also downregulated Cdc42 expression, but there was no significant difference. These investigations strongly indicated that DT could withhold the invasion of AM cells by suppressing Rho GTPases *in vivo*.

## 4 Discussion

AM, defined as the presence of endometrial stroma and glands in the myometrium, is a prevalent disease in women that manifests varying symptomatology and adverse health outcomes. Among these, pain plays a central role in patients with AM (Leuenberger et al., 2022). The management of AM is challenging and limiting. Most therapeutic drugs for relieving pain have been contraceptives, but symptoms recur in up to 75% of cases, making it a pressing need to find novel and safer therapeutic drugs.

Traditional Chinese medicine (TCM) has become a widely accepted alternative therapy to alleviate the discomfort of AM. According to the theory of TCM, “blood stasis” is the core pathogenesis of AM (Bi, 2020), resulting in the progressive exacerbation of dysmenorrhea in AM (Imanaka et al., 2021; Alcalde et al., 2022). TCM for promoting blood circulation has been shown to alleviate AM dysmenorrhea symptoms, reduce menstrual volume, and lower CA125 levels (Du and Du, 2020) (Zi et al., 2020) (Wang H Y et al., 2018). Dahuang - Taoren (DT), a classic botanical drug combination to combat “blood stasis,” is clinically used for AM treatment in China. In the study, the pain sensation in AM mice was dramatically relieved after DT treatment, indicating its promising effect on AM.

Though AM is benign, it shares certain characteristics with cancers, such as the ability to invade and metastasize. Growing evidence indicates that Rho GTPases, one of the most important regulators of cytoskeletal protein (Guan et al., 2020), play a crucial role in the abnormal motility of tumor cells (Kim et al., 2022; Qiu et al., 2022; Zhang et al., 2022). The Rho GTPases consist of 60 members and can be divided into multiple subfamilies (Jansen et al., 2018; Clayton and Ridley, 2020). RhoA, Rac1, and Cdc42 are the most well-studied members. It has been confirmed that the activation of RhoA and Rac1 enhanced the migration of colorectal cancer (Xie et al., 2021) (Wufuer et al., 2021). Other researchers have found that inhibition of Cdc42 was responsible for the suppression of invasion ability in lung cancer (Li et al., 2021), while the reduced RhoA had an anti-metastatic function on breast cancer (Kalpana et al., 2021).

Further research suggests that Rho GTPases directly affect the plasticity of cells and promote cell movement by remodeling the cytoskeleton. Thoroughly, the cellular microfilament skeleton dynamics are represented by a network of branching actin filaments gathering at the leading edge, pushing the membrane outward and generating the mechanical forces necessary for cell movement. Motile cell leading edges adhere to the extracellular matrix (ECM) and decompose focal adhesion, retracting in the tail subsequently, inducing cell motility. Cdc42, one of the Rho GTPases, can regulate filopodia formation for tracing cell movement. Once extracellular stimuli are detected, filopodia form on the cells to sense chemotactic signals. Although there is controversy about Cdc42 expression in AM disease (Goteri et al., 2006), our research showed that Cdc42 in the eutopic endometrium of AM was dramatically higher than that in the control group. It was suggested that overexpression of Cdc42 may lead to the formation of more filopodia (Marugg et al., 1990; Liu et al., 2019), and result in an aggressive chemotactic movement. Once the filopodia have determined their migration direction, a highly branched actin network, known as lamellar pseudopodia, is formed at the cell’s leading edge. This effect is regulated by Rac1, another kind of Rho GTPases. Rac1 mainly promotes the formation of lamellipodia and membrane ruffles (Guan et al., 2020). Rac1 and Cdc42 proteins share 72% sequence identity and thus have many same effectors (Humphries et al., 2020). For example, PAK1, an effector of Cdc42 and Rac1, was reported to be upregulated in AM (Zuo et al., 2019). In our research, the upregulation of Rac1 has also occurred in the eutopic endometrium of AM. Studies have confirmed that Rac1 and Cdc42 proteins can promote cell migration, proliferation, and invasion by regulating the cytoskeleton *via* activating phosphoinositide 3-kinase (PI3K) (Yan et al., 2018; Zhang et al., 2020). Therefore, it is speculated that high expression of Cdc42 and Rac1 may contribute to AM’s abnormal migration of endometrial cells.

RhoA, another classic Rho GTPases, is constantly enriched in the back of cells, responsible for retracting migrating cells’ tails (Hetmanski et al., 2019). Rock1 is the most detailed downstream effector molecule of Rho. RhoA/Rock1 can directly phosphorylate MLC and cofilin and then promote actin-myosin contraction (Xue et al., 2019). Besides, RhoA/Rock1 also leads to RhoA-mediated stress fiber formation (Totsukawa et al., 2000). It was reported that RhoA and Rock1 are highly expressed at the endometrial myometrium junction of AM and related to the abnormal contraction of smooth muscle cells (Wang et al., 2016). Other studies have reported that high expression of RhoA and Rock1 were positively associated with the severity of menorrhagia and menstrual volume in AM (Jiang et al., 2018). The activation of RhoA/Rock1 was considered to enhance the migration and proliferation of human eutopic endometrial epithelial cells (Huang et al., 2020). Consistently, our study found that the levels of RhoA and Rock1 in eutopic endometrium were upregulated compared with the control group.

It has been demonstrated that the treatment of TCM could downregulate the Rho/Rock1 (Zhang, 2014), RhoA, and Cdc42 (Sun et al., 2018; Guo et al., 2019), resulting in the inhibition of cell proliferation. It has been demonstrated that Rhein derivatives can strongly inhibiting breast cancer cell proliferation, migration, and invasion by inhibited Rac1 promoter activity and downregulated Rac1 protein expression (Li et al., 2020). Previous studies have demonstrated that emodin could inhibit RhoA and Rock1 on gene and protein levels, and it can also suppress the phosphatidylinositol 3-kinase-Cdc42/Rac1 pathway, resulting in the restrained migratory movement of tumor cells (Huang et al., 2005; Tan et al., 2020). In addition, rhein can inhibits the migration and invasion of cancer cells, *via* Rac1/ROS/MAPK signaling pathway (Zhou et al., 2017). Some researchers found that rhein can significantly reduce the abnormal endometrial proliferation of adenomyosis, in a dose-dependent manner, attributing to the suppressed Rac1 (Feng et al., 2017). Similarly, we found that DT could decrease the degree of pathological infiltration in AM mice, ameliorate the abnormal migration of endometrial stromal cells in a dose-dependent manner, companies with reduced expressions of RhoA, Rock1, Cdc42, and Rac1. Meanwhile, remodeling of the cytoskeleton was observed in AM mice (Ibrahim et al., 2015; An et al., 2017). The results of transmission electron microscopy in our research demonstrated that the glandular epithelial cells from the control group have a stable cell morphology structure, presenting finger-like structures and multiple tight junctions on the surface of the cell membrane, establishing a toughness and mechanical support between their cells. Conversely, the AM cells from the model group were elongated to fusiform, followed by decreased desmosome junctions and degraded finger-like structures, indicating high cell motility. The cell morphology structure was rebuilt after DT treatment, underlying its potential role in the cytoskeleton of the AM cell.

To sum up, the *in vitro* experiments demonstrated that DT could inhibit the migration and invasion of endometrial stromal cells in a dose-dependent manner by down-regulating RhoA, Rock1, Cdc42, and Rac1. Meanwhile, animal experiments confirmed that DT reduced myometrial infiltration, attenuated generalized hyperalgesia, and inhibited cytoskeletal remodeling by reducing Rho GTPase in AM mice.

## 5 Conclusion

Our study indicated that DT alleviated AM by inhibiting migration and invasion *via* suppressed Rho GTPases. It provides different insights into the pathogenesis of AM and the therapeutic mechanism of TCM.

## Data Availability

The original contributions presented in the study are included in the article/Supplementary Materials, further inquiries can be directed to the corresponding authors.

## Abbreviations

AM, adenomyosis; DT, Dahuang and Taoren; AMESCs, adenomyosis endometrial stromal cells; SPF, specific pathogen-free; ECM, extracellular matrix; PI3K, phosphoinositide 3-kinase; TCM, traditional Chinese medicine; HE, hematoxylin and eosin; TJ, multiple tight junctions.
