# Supplementary material for: Dahuang—Taoren, a botanical drug combination, ameliorates adenomyosis via inhibiting Rho GTPases
Source: Front Pharmacol. 2023 Mar 6;14:1089004. doi: 10.3389/fphar.2023.1089004 (PMC10035534; doi:10.3389/fphar.2023.1089004)
Supplement: Supplementary file 1 [file DataSheet1.docx]

**Supplementary Material** **S1**

**1.** **Preparation of Dahuang and Taoren** **extract granules**

Rinse and dry the crude drug, then cut it into slices. Add 6-8 times the amount of water into raw materials, and heat the raw materials at 100℃ for 3 hours under normal pressure. Then take out the first extract. Add 5-7 times as much water as the amount of raw materials into the first residue, and heat the residue for 2 hours at 100℃ under atmospheric pressure, then take out the second extract. The first and second extracts were combined and filtered, and the filtrated extracts were concentrated under pressure 0.02-0.09Mpa and temperature 80℃ and concentrated into fluid extract with a specific gravity of 1.20-1.35. The second residue was dried and crushed into a fine powder of the residue as the filling agent. The fine powder of pharmaceutical residue and the above fluid extract with a specific gravity of 1.20-1.35 are stirred and evenly mixed to form a mixture. Water vapor gas or nitrogen gas is used as atomizing gas for spray drying to produce extract granules of Dahuang or Taoren.

1g of Dahuang extract granules was extracted from 5g of crude Dahuang materials, and 1g of Taoren extract was obtained from 20g of crude Taoren materials.

The DT combination was prepared by mixing the extract granules of Dahuang and Taoren at the ratio of 4:1 (Each 5g DT extract granules mixture contained 4g Dahuang extract granules and 1g Taoren extract granules).

**2.Chemicals, materials and equipment for HPLC**

Dahuang granula(Approval no. 19004961) and Taoren granula (Approval no. 19006412) were purchased from Beijing Kangrentang Pharmaceutical Co., Ltd (Beijing, China).

Six standard substances aloe-emodin (batch No. 110795-201710), rhein (batch No. 110757-201607), emodin (batch No. 110756-201512), chrysophanol (lot No. 110796-201621), physcion (lot No. 110758-201621) were provided by the National Institutes for Food and Drug Control (Beijing, China), and the amygdaloside reference substance (batch No. K-001-171216) were offered by Chengdu Ruifensi Biotechnology Co., Ltd (Chengdu, China).

Series LC-2010A Liquid Chromatograph instrument (Shimadzu, Japan) was employed to analyze the extract solution. The chromatographic separation of Dahuang and Taoren Samples was performed using Dikmate (No. 20190125C18) and Hypersil (No. 20190224C18), respectively.

**3. Preparation of** **reference substance and DT sample for HPLC**

Dahuang standard stock solutions of aloe-emodin (50 µg/ml), rhein (50 µg/ml), emodin (50 µg/ml), chrysophanol (50 µg/ml) and physcione (25 µg/ml) were prepared in methanol. 2 ml of each of the above reference solutions was prepared and mixed well (1ml of Dahuang control solution contained aloe-emodin, rhein, emodin and chrysophanol 10 µg respectively and physcione 5µg). Amygdalin, used as quantitative stands of Taoren, was precisely weighted and diluted with 1ml of 70% methanol to 100µg/ml solution.

The Dahuang formula granules were pulverized into homogenous powder, and then the powder (0.15 g) was dissolved with 25 ml of 70% methanol proceed by heating reflux for 1h. After solvent volatilization, 10 ml of 8% hydrochloric acid was subjected to mix into 5 ml of filtrate, followed by sonication for 2 min and then by trichloromethane extraction. The trichloromethane layer, sprayed as a dry residue, was reconstituted in methanol for quantitative analysis (1 ml of sample solution for every 3 mg of extract granules).

Taoren granula (0.1 g) was reflux-extracted 50 ml of 70% methanol by conducting ultrasonic treatment (power 250 W, frequency 50 kHz) for 30 min. After filtration, 5 ml of filtrate was taken and added to 70% methanol at a constant volume to 10 ml for HPLC analysis (1 ml of sample solution for every 1 mg of extract granules).

**4. HPLC measuring**

The mobile phase of Dahuang was methanol-0.1% phosphoric acid aqueous solution (85: 15), and the procedure of gradient elution was as followed: 0-3 min 20%-30% methanol; 3-30 min 30%-45% methanol; 30-55 min 45%-70% methanol; 55-80 min 70%-80% methanol; 80-85 min 80% methanol; 85-90 min 80%-95% methanol; 90-95 min 95%-100% methanol; 95-100 min 100% methyl alcohol; column temperature: 30 ℃; sample size: 10 µl; flow rate: 1 min/ml; detection wavelength: 254 nm.

The mobile phase of Taoren was the methanol-aqueous solution (1: 4) and isocratic elution. The column temperature was 35℃, the flow rate was 1 ml/min, and the sample size was 10 µl. the wavelength was 210 nm.

**5. HPLC result**

The component content was investigated through retention time and area of the chromatographic peaks compare with the reference substance. Aloe-emodin (3.33 mg/g), rhein (3.33mg/g), emodin (3.33 mg/g), chrysophanol (3.33 mg/g), physcione (1.67mg/g) were identified in Dahuang, and amygdalin(100mg/g) were identified in Tanren from HPLC fingerprint respectively.

**TABLE S1** Retention time and Area (microvolts * second) of the six compounds of DT reference substance. Dahuang: 254 nm Taoren: 210 nm

| compound | retention time(min) | Area (microvolts *second) | Herb source |
| --- | --- | --- | --- |
| aloe-emodin | 6.169 | 8143.550 | Dahuang |
| rhein | 7.704 | 7467.349 | Dahuang |
| emodin | 12.190 | 9865.992 | Dahuang |
| chrysophanol | 16.820 | 12112.638 | Dahuang |
| physcione | 23.499 | 11833.638 | Dahuang |
| amygdalin | 10.513 | 1029171 | Taoren |

**TABLE S2** Retention time and Area (microvolts * second) of the six compounds of DT sample. Dahuang: 254 nm Taoren: 210 nm

| compound | retention time(min) | Area (microvolts * second) | Herb source |
| --- | --- | --- | --- |
| aloe-emodin | 6.179 | 8711.313 | Dahuang |
| rhein | 7.728 | 7538.207 | Dahuang |
| emodin | 12.228 | 9761.478 | Dahuang |
| chrysophanol | 16.858 | 12187.684 | Dahuang |
| physcione | 23.544 | 11816.460 | Dahuang |
| amygdalin | 10.592 | 851472 | Taoren |


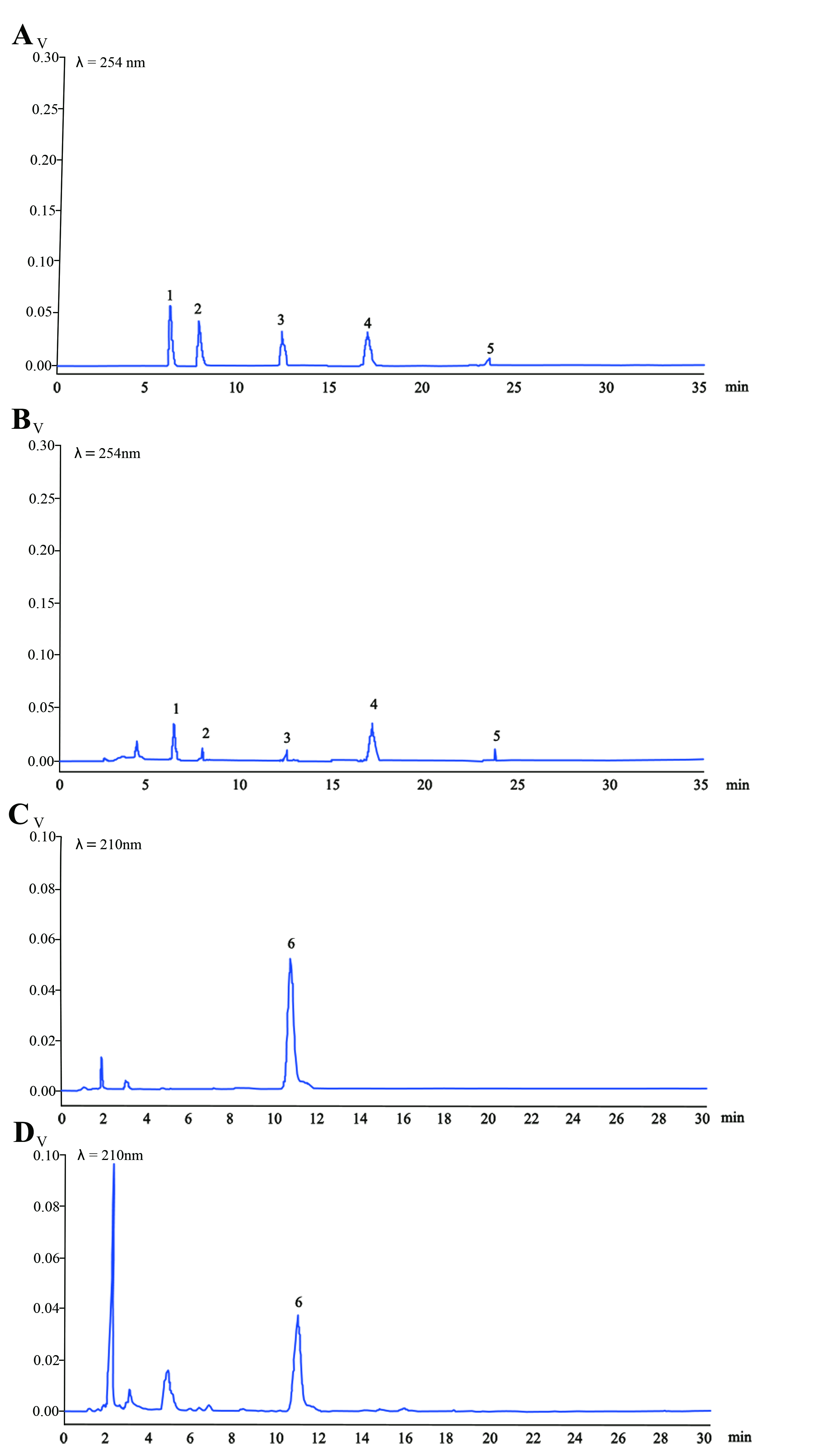


**FIGURE S1** HPLC chromatograms fingerprint analysis of DT granula. **(A)** Dahuang reference standard compounds. **(B)** Dahuang sample. **(C)** Taoren reference standard substance. **(D)** Dahuang sample. 1: aloe-emodin (3.33 mg/g), 2: rhein (3.33mg/g), 3: emodin (3.33 mg/g), 4: chrysophanol (3.33 mg/g), 5: physcione (1.67mg/g), 6: amygdalin(100mg/g)
